# A Systematic Review of the Legal Considerations Surrounding Medicines Management

**DOI:** 10.3390/medicina57010065

**Published:** 2021-01-13

**Authors:** Mojtaba Vaismoradi, Sue Jordan, Patricia A. Logan, Sara Amaniyan, Manela Glarcher

**Affiliations:** 1 Faculty of Nursing and Health Sciences, Nord University, 8049 Bodø, Norway; 2 Department of Nursing, Swansea University, Swansea SA2 8PP, UK; s.e.jordan@swansea.ac.uk; 3 Faculty of Science, Charles Sturt University, Bathurst 2795, Australia; plogan@csu.edu.au; 4 Student Research Center, Semnan University of Medical Sciences, Semnan 3514799442, Iran; s.amaniyan98@semums.ac.ir; 5 Institute of Nursing Science and Practice, Paracelsus Medical University, 5020 Salzburg, Austria; manela.glarcher@pmu.ac.at

**Keywords:** clinical practice, law, legal, adverse drug reactions, pharmacovigilance, medicines management, patient safety

## Abstract

*Background and Objectives*: There is a paucity of integrated knowledge regarding legal considerations required to ensure patient safety through safe medicines management. This study explores the legal considerations surrounding medicines management, providing a synthesis of existing knowledge. An integrative systematic review of the current international knowledge was performed. *Materials and Methods*: The search encompassed the online databases of PubMed (including Medline), Scopus, CINAHL, and Web of Science using MeSH terms and relevant keywords relating to the legal considerations of medicines management in healthcare settings. *Results*: The search process led to the identification of 6051 studies published between 2010 and 2020, of which six articles were found to be appropriate for data analysis and synthesis based on inclusion criteria. Research methods were varied and included qualitative interviews, mixed-methods designs, retrospective case reports and cross-sectional interrupted time-series analysis. Their foci were on the delegation of medicines management, pharmacovigilance and reporting of adverse drug reactions (ADRs) before and after legislation by nurses, physicians and pharmacists, medico-legal litigation, use of forced medication and the prescription monitoring program. Given the heterogenicity of the studies in terms of aims and research methods, a meta-analysis could not be performed and, therefore, our review findings are presented narratively under the categories of ‘healthcare providers’ education and monitoring tasks’, ‘individual and shared responsibility’, and ‘patients’ rights’. *Conclusion*: This review identifies legal aspects surrounding medicines management, including supervision and monitoring of the effects of medicines; healthcare providers’ knowledge and attitudes; support and standardised tools for monitoring and reporting medicines’ adverse side effects/ADRs; electronic health record systems; individual and shared perceptions of responsibility; recognition of nurses’ roles; detection of sentinel medication errors; covert or non-voluntary administration of medication, and patient participation.

## 1. Introduction

Health care is a complex system in which patients often experience harm resulting from the healthcare process itself [1,2]. It is too frequently accompanied by adverse events and medical errors [3], which are often preventable [4]. The World Health Organization (WHO) indicates that harm is caused in approximately one in 10 hospitalized patients, with at least 50% of these harms being preventable. Estimates show that 421 million hospitalizations take place worldwide every year and that during hospitalization about 42.7 million adverse events occur, and 18.3% of adverse events are attributed to medication errors [5]. A systematic review of 25 studies conducted in 27 countries showed that 2.9–21.9% of patients were affected by at least one adverse event, many of which were medication-related, and 34.3–83% could be prevented [6]. Beside deaths and serious adverse drug reactions (ADRs) in community settings, 128,000 patient deaths from prescribed medicines are reported annually in hospitals in the USA [7]. Therefore, measures for improving the quality of healthcare ensure or optimise patient safety “as the absence of preventable harm to a patient during the process of health care and reduction of risk of unnecessary harm associated with health care to an acceptable minimum” [8]. These efforts have gained critical international significance: in 2004, the founding of the World Alliance for Patient Safety by the WHO provided an initiative to focus on and improve the quality of care and patient safety. This alliance cooperates with health-related partners, e.g., with the 24 participating ministries of health or National Patient Safety Agencies, to achieve global improvement in healthcare, including the prescribing, dispensing and administering of medicines [9]. The European Commission (EC) intensified further developments to improve the safety of health care in Europe with the Luxembourg Declaration on Patient Safety in 2005. This indicates that 50% of all preventable adverse events are a consequence of ‘medication errors’, and has led to the development of specific recommendations to Europe-wide institutions, national authorities and healthcare providers [10,11]. 

### Medicines Management and Law

The overall goal of healthcare interventions is to ensure safe and high-quality patient care [12] through the safe and effective use of medications for the treatment of diseases [13]. There is a growing demand for the prescription of medications to treat age-related and chronic diseases worldwide. As an example, in Organisation for Economic Co-operation and Development (OECD) countries in 2017, the retail trade in pharmaceuticals accounted for one-fifth of all healthcare expenditures, averaging $564 per person [14]. Medicines have the ability to prevent, treat and cure diseases, but errors in the medication process that determines how medicines are used can cause damage. Therefore, the nature of pharmacotherapy demands that systems be in place to ensure the correct use of medicines, and that all transactions relating to medications be governed by appropriate laws and regulations [10]. 

According to the WHO Constitution (1946), “the highest attainable standard of health is a fundamental right of every human being”. Therefore, legal considerations are taken up by all countries through domestic or constitutional law to ensure access to high quality and safe health care [15]. It regulates behaviours or procedures that must be followed by individuals for maintaining human health, controlling or changing personal and professional behaviours [3]. 

Medicines management, as the handling of medications and medicinal products by healthcare professionals, consists of prescribing, dispensing, distributing, administration, patient education, follow up and monitoring, and is regulated by law [13,16]. In most cases, such laws contain a legal definition of a medicine, which also influences what can be purchased over the counter and when a prescription is required to obtain a medication. This provides a framework for governments to monitor the use of medications in the workplace, and defines how and according to which guidelines medications can be administered, including provisions for emergency situations [13]. Nonetheless, there are international differences in regulations for medicines management. In the United States, the Food and Drug Administration (FDA) of the U.S. Department of Health and Human Services pursues the protection of public health, which includes monitoring the safety and efficacy of medications. In many ways, the Drug Amendments of 1962 by the FDA is a model followed by other countries [17]. Within the European Union (EU), medicines law consists of European Commission directives and regulations that member states incorporate into national law [10]. 

Principles of medicines management developed based on laws and regulations should be unambiguously communicated through guidelines to healthcare professionals for indispensable use in clinical practice [18,19]. From the prescription to the administration of a medicine to a patient, physicians, nurses and pharmacists collaborate as a multidisciplinary team. However, problems can arise due to the unclear legal arrangements for medicines management [18]. Costs arising from additional hospital stays, litigation costs, hospital-acquired infections, loss of income, disability, and medical expenses have been reported as $6 to $29 billion annually [5]. 

Legal considerations to ensure patient safety through safe medicines management are therefore at the top of the political agenda, but there is a paucity of integrated knowledge. Accordingly, this systematic review of the international literature aims to answer the following question: what are the legal considerations for medicines management in healthcare settings? 

## 2. Materials and Methods

### 2.1. Design

A systematic review of literature was carried out as an explicit and objective research method for data collection and knowledge synthesis to reach our study aim [20]. An integrative approach to systematic reviewing was chosen, involving both qualitative and quantitative studies. It aims at summarising empirical or theoretical literature to provide a more comprehensive understanding of healthcare problems and can inform future research, clinical practice, and policy initiatives [21]. 

### 2.2. Search Method and Inclusion Criteria

The research protocol was developed (Appendix A) and three authors (MV, SA, MG) participated in the search process using four large online international databases that cover the majority of life sciences’ citations: PubMed (including Medline), Scopus, CINAHL and Web of Science. A reference librarian was approached to check the search process. The search aimed to retrieve articles published in English in the decade from 2010 to 2020. 

To maximise the number of studies retrieved, a pilot search in general and specialised databases, based on our experience of medicines management, was undertaken. This identified all MeSH terms and all relevant keywords relating to the ‘legal considerations’ for ‘medicines management’. Boolean operators (AND, OR) were used to build search phrases and search titles and abstracts. Grey literature on policy documents, clinical guidelines and cross-references from bibliographies were used to improve the search coverage. 

Inclusion criteria were empirical studies with a focus on legal considerations of medicines management in clinical practice, in short-term and long-term healthcare settings, and published in online peer-reviewed scientific journals. Thus, reviews, commentaries, letters, conference proceedings, and those studies with a concentration on medicines management in places other than healthcare settings were excluded. The authors independently screened the titles, abstracts, and full texts of the retrieved studies, held discussions, and reached agreement regarding the inclusion of selected studies based on the inclusion criteria. 

### 2.3. Quality Appraisal 

Appropriate tools to the selected studies’ methods were used, including the Strengthening the Reporting of Observational Studies in Epidemiology (STROBE) (for cross-sectional studies, maximum score 30) [22], the Critical Appraisal Skills Programme (CASP) for qualitative studies (maximum score 10) [23], Mixed Methods Appraisal Tool (MMAT) (maximum score 17) [24], and Joanna Briggs Institute (JBI) Critical Appraisal Checklist for Case Reports (maximum score 8) [25]. Overall, the final decision on the importance and methodological quality of each study for inclusion or exclusion was made after holding discussions between the authors and reaching agreement. 

### 2.4. Data Abstraction and Knowledge Synthesis

A pre-piloted data extraction table was developed to import data from selected studies and categorise them according to: author’s name, publication year, country, design, sample size and setting, and main findings concerning legal aspects of medicines management in healthcare settings. Before the full data extraction, the table was pilot-tested to ensure that it suited the review aim by including the required data for knowledge analysis [20]. 

Variations within the selected studies in terms of aims and methods precluded a meta-analysis. Therefore, the review findings are presented narratively based on the Preferred Reporting Items Systematic Reviews and Meta-Analysis (PRISMA) statement [26]. Categories were developed by the authors based on differences and similarities in the studies’ findings. The authors undertook discussions to reach agreements on assigning the studies’ findings into the categories. 

## 3. Results

### 3.1. Search Results and Study Selections

**Figure 1:**
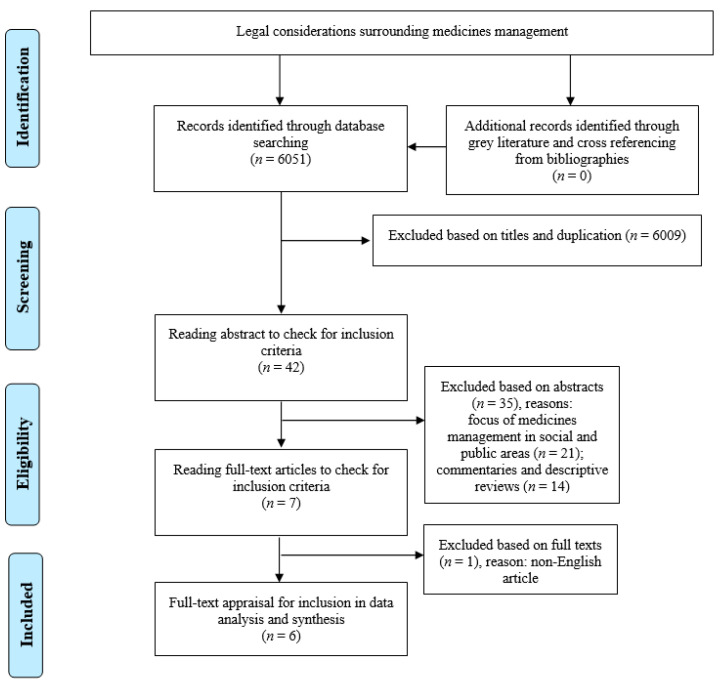
The process of the review adapted from the Preferred Reporting Items for Systematic Reviews and Meta-Analysis (PRISMA) [26]. The PRISMA Statement distributed under the terms of the Creative Commons Attribution License.

The literature search in the databases led to the retrieval of 6051 articles in total from four databases (Figure 1). Deletion of duplicate titles and irrelevant studies based on independent title and abstract reading by the authors led to a final selection of 42 studies. They were carefully checked through abstract-reading with an outcome of seven studies fully meeting the inclusion criteria, which were selected for full-text reading and appraisal (Table 1). Excluded studies discussed the social aspects influencing medication rather than legal considerations in healthcare settings or had no exact relevance to patient-safety principles. 

The full-texts were obtained from the Norwegian library and were carefully read in order to select only those studies with a precise focus on the legal considerations of medicines management and related factors in healthcare settings, and those with a high-quality method in their research processes based on scores achieved using the appraisal tools. The full-text of one article was in French and was thus excluded, leaving six studies for full-text appraisal. The remaining articles (*n* = 6) were relevant to the review topic and had acceptable research structure and framework quality (Table 2). No further studies were found for inclusion during the grey literature and manual search in the reference lists of the selected studies.

### 3.2. General Characteristics of the Selected Studies

Table 2 provides an overview of the general characteristics of the selected studies for data analysis and synthesis. The studies were published between 2013 and 2019 and were conducted in Sweden [27,29], Portugal [28], Italy [30], Denmark [31] and USA [32].

There were variations in the studies’ methods and research foci. One study comprised qualitative interviews focusing on nurses’ perspectives of delegation of medicines management to unlicensed/ unregistered staff [27]; another was a mixed-methods study consisting of survey and focus groups with pharmacists regarding ADR reporting and knowledge of pharmacovigilance legislation [28]; two studies were retrospective case reports of medico-legal litigation and the management of clinical risk [30] and legal aspects of patients’ rights in forced medication orders [31]; one was a cross-sectional study of ADR reporting before and after legislation changes and the conditions for nurse reporting [29] and another was a cross-sectional interrupted time-series analysis of prescription drug-monitoring programs and the related patient risk [32].

### 3.3. Legal Considerations of Medicines Management

**Figure 2:**
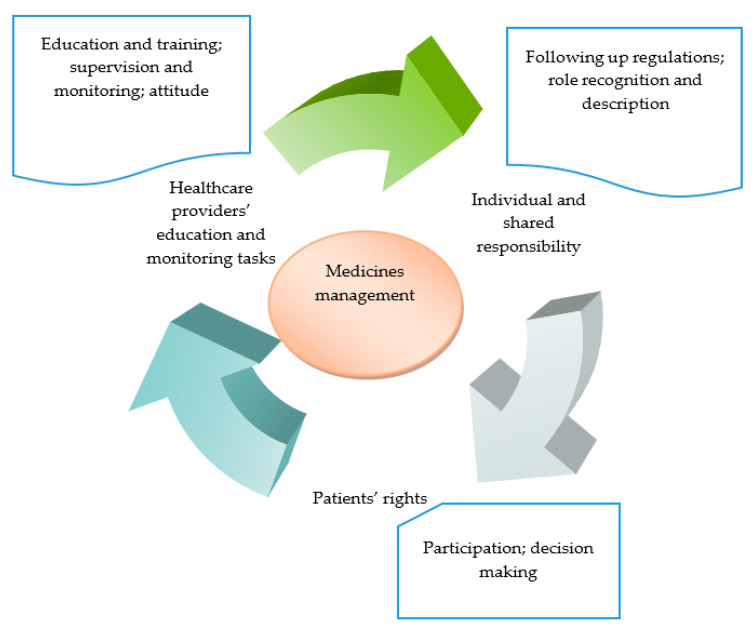
Categories developed regarding legal considerations surrounding medicines management.

The review findings were classified and grouped, based on the similarities and differences between the findings of the selected studies. The classification highlighted the legal considerations affecting medicines management in healthcare settings within the following categories developed by the authors: ‘healthcare providers’ education and monitoring tasks’, ‘individual and shared responsibility’ and ‘patients’ rights’. They highlighted the interconnections of the legislation concerning medicines management, intended to improve the quality and safety of the medication process (Figure 2).

Given these heterogeneities in the studies’ methods, objectives and results, we presented the results of this review narratively under categories developed based on similarities and differences between the studies’ findings. A summary of the studies’ results has been presented in Table 3.

#### 3.3.1. Healthcare Providers’ Education and Monitoring Tasks

A series of measures to support the lawful practice of medicines management including the education of healthcare staff and the application of monitoring tools was needed to guarantee the implementation of regulations and rules determined by legislation, with the aim of ensuring safe medicines management.

The education and empowerment of healthcare professionals was emphasised, based on findings that healthcare staff frequently lacked knowledge and skills regarding medicines management and its legal aspects.

Craftman et al., concluded delegation of medicines management to unlicensed healthcare staff could be lawful following sufficient education and training to prevent medication errors. Also, nurses who gained their knowledge of medicines management through practical and on-the-job training, rather than formal education, were identified as needing education and supervision to prevent errors during the preparation of medicines, mixing medicines, and crushing tablets before administration [27].

Duarte et al. indicated that not all pharmacists (70%) knew about the new pharmacovigilance legislation supporting the reporting of ADRs and side effects. Some lacked sufficient knowledge on the new definition of ADRs or lacked positive attitudes towards checking details on the relevant websites to be able to report suspected ADRs. Therefore, knowledge development in pharmacovigilance, clinical pharmacology and attribution of causality for medicines’ side effects and ADRs seemed necessary [28].

Legally enforced and standardised monitoring tools would ensure reporting medicines’ side effects and ADRs.

Karlsen et al., stated that before the passing of legislation supporting ADRs reporting in 2005, only 100 reports were submitted (ever), compared with 172 reports after legislation came into effect in 2010, and its implementation was monitored and supported [29].

According to Strickler et al., legal sanctions that addressed over-prescription and risky opioid use, together with the implementation of monitoring programs for the prescription of medicines, encouraged prescribers to actively follow medicines management guidelines and ensure the safety of medication through efforts to customize the law and suit it to cultural contexts that improved its effectiveness. The review and registration of the patient’s prescription history as a monitoring tool before prescribing opioids was mandated. This reduced multiple prescribing, the opioid prescribing rate, overlapping opioid prescriptions, and overlapping opioid/benzodiazepine prescriptions [32].

From the perspective of Craftman et al., standardised methods for the delegation of medicines management consisting of validated tools and guidelines were key to assess healthcare staff’s readiness for assuming responsibility for medication and risk management under the supervision of a licensed nurse [27]. Also, Strickler et al., believed that the use of the electronic health record system and customising the law to suit the care context in terms of staffing pattern and equipment helped with monitoring the medication process and reducing the risk of medication errors, and guaranteed the implementation of legal initiatives of medicines management [32].

#### 3.3.2. Individual and Shared Responsibility

The success of legal initiatives supporting medicines management depended on the creation of a sense of both individual and shared responsibility for the accurate implementation of the law. Legislation and enforcement actions could not guarantee adherence to medicines management guidelines. Legal initiatives alone were insufficient: shared motivation and responsibility were needed.

Craftman et al. reported that difficulties in following rules and regulations for delegating medication duties to unlicensed healthcare staff, combined with incompatibility between the regulations and current practice, detracted from individual responsibility. In general, regulations and guidelines were outdated and did not consider staff shortages, heavy workloads and transitional care from healthcare settings to patients’ own homes, causing a disconnect with current practice and changes [27]. In Duarte et al., whilst 66% of pharmacists were willing to report ADRs, difficulties related to the process of reporting, including malfunctioning websites and complicated and long reporting forms, were barriers to individual responsibility for reporting [28].

Duarte et al. defined shared responsibility for medicines management in terms of reporting and publishing reports of medicines’ side effects and ADRs by the entire chain of stakeholders, from the pharmaceutical industry to healthcare settings. Legislation should recognise the role of each healthcare worker and describe how each role is responsible for safe medicines management [28]. Craftman et al. also stated that the duty of the delegation of medication by nurses to other healthcare staff should be legally recognised and supported in order to prevent interference in other healthcare staff’s responsibilities and damaging their feeling of accountability for medicines management in community settings [27]. Karlsen et al., found that after the introduction of legislation on reporting ADRs in 2010, physicians more often reported (75%) than nurses (24%). Reporting of serious ADRs stood at 3–7% and 48–49% by nurses and physicians, respectively. These reports were mostly related to systemic antibiotics, vaccines and antivirals, nervous system drugs, contrast media, and respiratory medications. The reduction in the number of ADR reports by nurses after legislation introduction was attributed to inappropriate definition of nurses’ roles in ADR reporting and restriction of their scope of practice in reporting ADRs to a narrow subset of medications. The close contact between nurses and patients and their general responsibility for medicines administration and assessment of their outcomes in hospitalised patients highlighted nurses’ valuable role in improving pharmacovigilance, but this role was not clearly specified in law and so failed to support nurses as ADR reporters [29].

#### 3.3.3. Patients’ Rights

Medication practice with appropriate legal oversight supported patients’ rights through: selection of the most effective medicines with the lowest risks of adverse side effects, patient participation as the provision of choice between medications and information on probable side effects and adverse reactions.

Bolcato et al. stated that sentinel cases of medication errors are unanticipated events, unrelated to the patient’s underlying health condition, resulting in death or serious physical or psychological damage. Sentinel medication errors during a 2-year period arising in the course of medicines management had legal consequences, including compensation. Events included: botulinum injection during surgery for mild equinism on the wrong foot; no prescription of low molecular weight heparin after cardiac surgery followed by massive pulmonary embolism; and frequent intra-detrusoral infiltration of botulinum for recurrent cystitis, followed by bladder dysfunction and sexual impairment, of which eight cases were compensated and 15 cases were under treatment [30].

Gøtzsche et al. highlighted the issues of ignoring the law and patients’ rights during the medication process: 30 consecutive appeals over the forced use of mental health medicines were reported. They included: failure to assess patients’ medical files, absence of expert consultation regarding the necessity of prescriptions, selection of medicines with the highest risks of adverse effects (olanzapine, risperidone, zuclopenthixol, paliperidone, quetiapine, aripiprazole), injection, rather than oral administration, exceeding the manufacturers’ recommended doses, failure to attempt patient participation and motivate the patient to accept treatment voluntarily before forced use, and lack of documentation for forced prescriptions and the patient’s inability to give informed consent [31].

## 4. Discussion

This systematic review, through integrating the findings of empirical qualitative and quantitative studies, augments our knowledge regarding the legal considerations for medicines management in healthcare settings.

Measures to guarantee the implementation of legal initiatives and regulations for safe medicines management depended on the education of healthcare staff and monitoring the implementation of safety regulations. Unmet educational needs in pharmacology and medicines management, as well as ineffective evaluation of medication practice, represent common issues affecting patient safety across healthcare disciplines [33]. Knowledge of the law and legal considerations surrounding medicines management is often under-represented in healthcare curricula [34]. However, the development of medicines management competencies requires appropriate clinical education and supervision [35,36] and should encompass the appropriate use of standard checklists and the development of action plans to remove the gap between policymaking and implementation in practice [37]. Standardised safe medication checklists as monitoring tools have been proven to be effective in preventing lapses and reducing the incidence of adverse events by ensuring the implementation of the law and adherence to related guidelines [38].

This review highlighted the need for standardised tools to guide the implementation of legal initiatives and facilitate the participation of healthcare providers and patients in medicines management. Structures and processes that standardise care are key to preventing medication errors [39]. Surveillance systems should encompass all aspects of the medication cycle, including prescribing, distribution, administration and monitoring [40]. Systematized, formal and documented processes help to identify and address medication errors and adverse events while they are still containable, and before harm occurs to patients [19,41]. Risk assessment tools and corresponding standards, and/or national guidelines for monitoring and handling certain high-risk medications increase the safety of administration, and similar process should be applied to encourage reporting of errors [42,43].

Our review indicates that reporting of ADRs is suboptimal and would be improved by a more robust legal framework. An ADR is an unintended and harmful reaction in the patient to a medicine associated with any administered dose, which can lead to life-threatening conditions, persistent disability, hospitalization, and even a patient’s death [44,45]. Under-reporting of ADRs has been attributed to limited knowledge among healthcare providers and limited durability of related educational interventions [46]. Additionally, poor awareness of the risks of under-reporting, particularly underestimation of these risks, inappropriate reporting tools, delayed or no feedback on reported ADRs, and fear of legal liability have been mentioned as common barriers to reporting ADRs [47]. Most reporting systems and electronic medical records contain no specific category for ADRs [48]. Training activities to rationally prescribe, distribute, and monitor medications with close follow-up for adverse reactions, and reporting mechanisms, are the main steps to improving pharmacovigilance [49,50,51]. Although national error reporting systems exist in many countries, the number of ADRs reported remains low and the quality of reports is often poor [19,48]. An intervention programme using the incorporation of legal aspects of medicines management and education via posters, lectures and electronic distance learning can enhance the knowledge and attitudes of caregivers towards reporting [19,52,53].

According to the review findings, the prescription and administration of medications should be directly addressed by the law. The presence of the law unifies the area of accountability among healthcare providers, allowing patients to benefit from medicines’ therapeutic effects as well as protecting them against medications’ harmful effects [53]. Clear laws and regulations for medicines management are the required underpinnings for clinical practice guidelines, which support safe and effective handling of medicines by healthcare providers [54]. Medication errors happen often when healthcare providers have insufficient knowledge about rules, modify rules or do not follow them in full [55]. Therefore, regular education to update knowledge and understanding of the medico-legal aspects of patient care is required to ensure quality of care [56]. All healthcare professionals have a duty to follow rules and regulations for safe medication practice. If these cannot be followed because of systemic deficiencies, professionals are obliged to report system shortcomings and suggest remedies [57]. It is thus the responsibility of healthcare managers to ensure that safety systems are in place and to ensure patient safety through the consideration of legal, regulatory, ethical, humanistic and practical considerations in addressing medication adverse events [58].

Incorporating medicines management programs into the electronic health record system was found to be important. Structured medication interventions using computerized decision support systems improves the appropriateness and accuracy of medication regimens among hospitalized patients [59]. In spite of current shortcomings in updated protocols for new medications, the use of electronic systems for medication prescription may improve patient safety through enhancing interprofessional communication and accountability [60]. For example, the use of digital devices that remind patients to take a pill, verify the actual intake, and collect and send related data to a remote computer system have been helpful. However, this raises questions about patients’ rights to autonomy and potentially violates privacy rights through the secondary use of patient data and healthcare providers’ data, which has implications for liability [61,62].

According to this review, individual and shared responsibility were required for the successful implementation of legal initiatives supporting medicines management. However, barriers to shared responsibilities included: the lack of knowledge of ADRs and reporting systems, incompatibility between the law and the healthcare context, and lack of recognition of healthcare staff roles in medicines management. While there is no consensus about which healthcare profession is most suited to medicines management roles, it is accepted that trained and competent staff should assume these critical roles [63,64]. Shared decision making as a means of acknowledging power differentials and providing information about medicines should be routine in all areas of health care, but this aspect of medicines management is under-represented in existing literature [65]. Since medication errors involve different healthcare professionals, a collaborative approach, especially among vulnerable patients, has been suggested [66]. Collaboration through communication, sharing information and the provision of regular feedback can improve adherence to the principles of safe medication practice [39]. Nurses have the required knowledge and skills regarding medicines management and spend more time with patients than physicians and pharmacists, increasing their chances of detecting medicines discrepancies and near misses [67,68,69]. Therefore, nurses’ role, accountability and knowledge of medications should be taken into account when strategies are devised to improve medicines management [19,66,70].

In our review the law should support how to detect near misses and medication errors with a sentinel identity, help prevent patient harm and reduce its impact on patients. Medication errors with serious consequences for the patient health often remain under-reported [71]. Disclosing medication errors through the regular use of audit and failure mode, effect, and criticality analysis (FMECA) improves the performance of individuals and the reliability of healthcare systems [72]. Learning from near misses and errors improves the culture of safety [66,73].

Medication practice should consider patients’ rights through the prescription of medicines with the fewest side effects, and patient participation by informing them of all possible adverse or undesirable effects. Patients’ health and well-being depend on collaboration between patients and healthcare providers in a respectful alliance. Healthcare providers serve patients as their advocates and respect their rights by providing them with the decision-making capacity to be able to accept or refuse recommended medications [74]. Disguising medicines in food or drink is a common practice (43–71%) in the majority of nursing homes [75] and is accompanied with incomplete documentation and consultation with patients’ representatives or other healthcare providers, contravening the law [75,76]. The preservation of patients’ rights in the contemporary healthcare system is more complicated than the linear process of medication administration and should consider the whole process of medicines management [77,78]. Patients’ rights should encompass the discussion of specific risks and benefits of proposed therapy with patients or their guardians and the documentation of informed consent to medicine administration in the medical record [79].

Limitations of this review include the heterogeneity of the selected studies’ methods and variations in their focus, which could impact the integration of the findings. A thorough search process using multidimensional keywords applied commonly in the field of healthcare was performed in international databases, but a few empirical studies were retrieved, indicating a paucity of research on this important aspect of patient safety. Despite the possibility of unintentional oversight by the authors in identifying all relevant keywords for the search, the review provides a contemporary overview of the legal considerations of medicines management as well as offering leads regarding aspects in need of further elaboration in future studies.

## 5. Conclusions

The findings of this review identified significant individual and system factors regarding the legal considerations of medicines management in the healthcare system. A summary of the review findings and suggested improvement strategies has been presented in Table 4.

These findings can be used for developing a framework of action to improve the safety of medicines management and avoid legal issues affecting both the healthcare provider and the patient.

## Figures and Tables

**Table 1:** The search strategy and results of different phases of the review.

Keywords Used for Search	Databases from 2010–2020	Total in Each Database	Selection Based on Title	Selection Based on Abstract	Selection Based on Full-Text Appraisal
(medication OR drug OR medicines OR “pharmaceutical preparations” OR pharmaceuticals OR “medicines management”) AND (law OR rule OR regulation OR principle OR legislation OR Act OR guideline OR legal OR bill OR convention OR policy OR obligation OR “breach of duty” OR “legal duty” OR accountability OR responsibility OR “patient’s right” OR litigation OR duty)	PubMed (including Medline)	2953	11	0	0
	Scopus	1336	11	2	3
	Web of Science	1460	14	2	2
	Cinahl	302	6	3	1
	Manual search/backtracking references	0	0	0	0
	Total of databases	6051	42	7	6

**Table 2:** Characteristics of selected studies for data analysis and synthesis.

Author, Year, Country	Aim	Methods	Sample and Setting	Main Finding	Conclusion	Full-Text Appraisal Score
Craftman et al., 2013, Sweden [27]	To explore nurses’ perspectives on the delegation of medicines management to unlicensed staff	Descriptive qualitative	20 nurses in district healthcare centres	Incompatibility of delegation with clinical practice; being in access to home care providers	Necessity of the delegation of medicines management and difficulty in performing the delegation task	9 out of 10
Duarte et al., 2015, Portugal [28]	To investigate ADRs reporting and knowledge of pharmacovigilance legislation among pharmacists	Mixed-methods consisting of (i) survey and (ii) focus group interview	(i) 154 pharmacists in the south of country; (ii) 7 community pharmacists	4965 and 5159 reports in 2005 and 2010 were submitted, respectively: (i) One-quarter were familiar with ADRs reporting; (ii) 38.3% reported ADRs, underreporting being due to attitude issues and lack of knowledge of casual relationship between ADRs and medicines	Need for education and training	14 out of 17
Karlsen et al., 2015, Sweden [29]	To understand ADRs reporting before and after legislation changes and the condition of nurses’ reporting	Cross-sectional	Registered reports in six regional centres for handling spontaneous ADRs reporting	898 and 1074 reports to the pharmacovigilance system in 2005 and 2010, respectively; 31% and 24% of reports made by nurses in these years	Increase in the number of serious/unlabelled ADRs reports after legislation	22 out of 30
Bolcato et al., 2019, Italy [30]	To investigate medico-legal litigation in the management of clinical risk and propose a model	Retrospective case report	206 cases of medico-legal litigation settled in an urban hospital from 2014–2015	20% of the cases remained unreported due to the latency between the event and its manifestations and discomfort in reporting	Need for the establishment of a model for rapid reporting	6 out of 8
Gøtzsche et al., 2019, Denmark [31]	To investigate if the law and patients’ rights were respected when forced medication orders were appealed	Retrospective case report	30 consecutive cases registered on the psychiatric appeals board	No clear indication of the suitability of treatment to the patient’s interest; violation of the law on the use of forced medications with the lowest adverse effects (97%)	Abandonment of forced medication use	6 out of 8
Strickler et al., 2019, USA [32]	To examine the effect of prescription drug monitoring programs on prescriber registration, the programs’ use and prescription-based measures of patient risk	Cross-sectional interrupted time-series analyses	Prescription and prescriber usage of the prescription drug monitoring programs from 5 states	Legal mandates increased the prescription and use of the programs; reduction of the multiple provider episode rate, opioid prescribing and overlapping, opioid/benzodiazepine prescriptions’ overlapping	Reduction of risky opioid prescriptions through prescription drug monitoring programs’ mandate	24 out of 30

ADRs: adverse drug reactions.

**Table 3:** Summary of review findings based on the three categories evident.

Categories	Healthcare Providers’ Education and Monitoring Tasks	Individual and Shared Responsibility	Patients’ Rights
Author, Year			
Craftman et al., 2013 [27]	Supervision, education and training regarding medication preparation and administration; validated guidelines and tools	Difficulty in following up regulations; recognition of the nurse’s role	No data
Duarte et al., 2015 [28]	Being informed and educated about pharmacovigilance; attitudes towards reporting	Difficulty in the process of reporting medicines’ side effects/ADRs; recognition of professionals’/employees’ roles	No data
Karlsen et al., 2015 [29]	Legislative support for reporting medication side effects and ADRs	Recognition of the nurse’s role	No data
Bolcato et al., 2019 [30]	No data	No data	Sentinel medication errors, their detection and compensation;
Gøtzsche et al., 2019 [31]	No data	No data	Patient participation, decision making for prescription and administration
Strickler et al., 2019 [32]	The use of the electronic health record system; customising the law	No data	No data

ADRs: adverse drug reactions.

**Table 4:** Improvement strategies based on the review findings.

Categories	Areas of Interest	Improvement Strategies
Healthcare providers’ education and monitoring tasks	Insufficient knowledge and skills regarding medicines management, particularly amongst unlicensed healthcare staff	Education and training regarding medicines preparation and administration; Direct supervision to ensure medicines management competencies
	Lack of knowledge of and negative attitudes towards pharmacovigilance legislation	Education on legislation influencing medicines management; Improvement of culture surrounding patient safety; Development of knowledge about the causes of medicines’ side effects and ADRs
	Effectiveness of legal monitoring guidelines to reduce medicines’ over-prescription and risky use	Application of standardised and contextually adapted checklists for safe medication practice
	Significance of validated tools for risk assessment and risk management during the medication process	Use of monitoring tools by healthcare leaders to monitor healthcare staff skills and healthcare environment to reduce errors and manage risks
	Highlighting the role of electronic health record systems	Application of electronic health record systems for the communication and documentation of the medication process; Use of computerized medication decision support systems
Individual and shared responsibility	Difficulties in following rules and regulations	Improvement of working conditions; Development of structured surveillance systems for prescribing, administration, and monitoring
	Importance of individual motivation to follow regulations	Supporting ADR reporting and following up medicines management guidelines
	Significance of healthcare provider’s roles and mutual reasonability towards safe medication practice	Education and training regarding the professional roles and duties of healthcare staff; Development of cultures of shared decision making; Collaboration through communication and mutual feedback; Clear task assignment and job description emphasising mutual responsibility towards safe medicines management
Patients’ rights	Need for the detection, reporting of sentinel medication errors and their appropriate compensation	Use of active error detection methods, analysis of and learning from potential medication errors
	Highlighting patients’ participation throughout the medication process	Informing the patient about risks and benefits of medicines; Inviting the patient to be involved in decision making regarding the selection of medicine

ADRs: adverse drug reactions.

## Data Availability

All data is contained within the article.

## Appendix A

**Table A1:** The protocol for the systematic review of legal considerations surrounding medicines management in healthcare settings.

Aspects and Definitions		Practical Notes
Identification of the research question	A systematic review on legal considerations of medicines management in healthcare settings	Legal considerations and strategies to ensure patient safety through safe medicines management influencing on the quality care in the healthcare system.
Review type	An integrative systematic review	Both qualitative and quantitative studies will be considered. Studies will be experimental, cross-sectional, qualitative, but reviews, commentaries, letters, and books will be excluded.
Language	English	
Study designs	All original research-based studies	
Literature search	Researcher(s)	An international team of researchers working on medicines management.
	Databases	Electronic databases such as Scopus, PubMed [including Medline], Cinahl, Web of Science for retrieving studies published from 2010 to 2020.
	Manual search and grey literature	Important journals for manual search will be identified during the references checking of retrieved articles selected for full-text appraisal. Grey literature includes policy documents, clinical guidelines, and cross-references from bibliographies for improving the search coverage.
	Keywords	After a pilot search in databases, keywords for search to be used via the Boolean Method are as follows: (medication OR drug OR medicines OR “pharmaceutical preparations” OR pharmaceuticals OR “medicines management”) AND (law OR rule OR regulation OR principle OR legislation OR Act OR guideline OR legal OR bill OR convention OR policy OR obligation OR “breach of duty” OR “legal duty” OR accountability OR responsibility OR “patient’s right” OR litigation OR duty).
Literature selection	Selecting original studies in stages	Based on titles, abstracts and full texts of articles.
	Pre-tested inclusion criteria for original studies	Explicit interest to the study topic; original and scientific content related to the study topic; focused on the legal considerations of medicines management in clinical practice in short-term and long-term healthcare settings and published in online peer-reviewed scientific journals. Articles without an exact relevance on legal aspects and considerations of medicines management in clinical practice will be excluded.
	Quality criteria	Methodological checklists for full-text appraisals can be found at: https://www.equator-network.org/
Equator guidelines	PRISMA	The whole process of review will be assessed using the methodological checklist of systematic reviews and meta-analyses developed by the http://prisma-statement.org/prismastatement/Checklist.aspx

PRISMA: Preferred Reporting Items Systematic Reviews and Meta-Analysis.
